# Effects of a Dietary Supplement Composed of Baicalin, Bromelain and Escin for Venous Chronic Insufficiency Treatment: Insights from a Retrospective Observational Study

**DOI:** 10.3390/ph17060779

**Published:** 2024-06-14

**Authors:** Selene Francesca Anna Drago, Michelangelo Rottura, Antonino Molonia, Viviana Maria Gianguzzo, Giovanni Pallio, Natasha Irrera, Luana Orlando, Marianna Gigliotti De Fazio, Marilena Isgrò, Natalia Zirilli, Vincenzo Arcoraci, Egidio Imbalzano

**Affiliations:** 1 Department of Clinical and Experimental Medicine, University of Messina, Via C. Valeria, 98125 Messina, Italy; selenefrancescaanna.drago@unime.it (S.F.A.D.); mrottura@unime.it (M.R.); anmolonia@unime.it (A.M.); nirrera@unime.it (N.I.); luana_orlando@libero.it (L.O.); mariannagdf89@hotmail.it (M.G.D.F.); marilenait90@outlook.it (M.I.); zirilli.natalia@hotmail.com (N.Z.); eimbalzano@unime.it (E.I.); 2 Department of Chemical, Biological, Pharmaceutical and Environmental Sciences, University of Messina, 98166 Messina, Italy; vivianamaria.gianguzzo@unime.it; 3 Department of Biomedical and Dental Sciences and Morphological and Functional Imaging, University of Messina, 98125 Messina, Italy; gpallio@unime.it

**Keywords:** chronic venous insufficiency, dietary supplement, baicalin, bromelain, escin, graduated compression stockings

## Abstract

Chronic venous insufficiency (CVI) represents a risk factor for cardiovascular events. The first-line treatment includes the use of compression stockings and lifestyle changes. Natural products, such as flavonoids, could be used to improve the effects of compression therapy due to their anti-inflammatory and anti-oxidant properties. This study aims to evaluate the effects of a dietary supplement containing baicalin, bromeline and escin in CVI patients. A retrospective cohort study was performed by using the medical records of CVI affected outpatients. Patients treated with the dietary supplement were defined as “users”. A modified Venous Clinical Severity Score (VCSS) was calculated, including pain, inflammation, vessels induration and skin pigmentation. All clinical variables were evaluated at baseline (T0), after 30 (T1) and 90(T2) days in “users” and “non-users”. Out of 62 patients, 30 (48.4%) were “users”. No difference was observed between groups at baseline. A lower VCSS value was recorded in “users” than that observed in “non-users” at T2 (7.0 (4.0–9.0) vs. 9.0 (5.0–10.0); *p* = 0.025). Vessels’ induration and pain significantly reduced in 53.3% and 43.3% of “users” and in 18.8% and 9.4% of “non-users”. Only “users” (33.3%) showed a reduction of the inflammatory signs as well as a decrease in malleolar circumference, from 29.0 (26.5–30.0) to 27.5 (26.0–28.5) (*p* < 000.1). A reduction of C-reactive Protein levels was found in “users” compared to “non-users” at T2 (1.0 (0.9–1.2) vs. 1.3 (1.0–1.5); *p* = 0.006). These findings suggest that implementation of a dietary supplement could improve the clinical outcomes of CVI patients.

## 1. Introduction

Chronic venous insufficiency (CVI) is a debilitating condition affecting millions of people worldwide; the pathophysiology is complex and still needs to be clarified, even if recent findings suggest that genetic and environmental factors might contribute to its development [1]. CVI is characterized by morphological and functional anomalies of the venous system and represents a risk factor for cardiovascular events. The most common symptoms fall under the category of subjectivity, which include discomfort, pain and leg heaviness; CVI clinical signs include varicose veins, edema, skin discoloration, lipo-dermatosclerosis up to chronic leg ulcer and deep vein thrombosis in severe cases. Moreover, age may worsen signs and symptoms, thus impacting the quality of life of the affected patients [2,3,4].

The Clinical–Etiology–Anatomy–Pathophysiology (CEAP) classification is a standard used for classifying chronic venous disorders. The chronic venous disorders are classified from C0 to C6 based on the severity of venous symptoms [5]. While the CEAP classification system is useful to classify the stages of venous disease, the Venous Clinical Severity Score (VCSS) is used to stratify disease severity. The VCSS includes 10 clinical descriptors (pain, varicose veins, venous edema, skin pigmentation, inflammation, vessels induration, number of active ulcers, duration of active ulceration, size of ulcer, and compressive therapy use) and is useful for evaluating changes in severity of disease over time with or without intervention [6]. Early action by healthcare professionals (HCPs) is crucial to prevent debilitating complications; nonetheless, patients generally do not seek treatment until cardiovascular disease (CVD) symptoms appear.

The European Society for Vascular Surgery (ESVS) has established guidelines for the management of CVI patients in clinical practice. The management of CVD-affected patients aims at alleviating venous symptoms and signs, stopping inflammation and preventing progression into a higher CEAP clinical class. Early intervention, including recommendations for lifestyle changes (e.g., loss of weight, physical activity, wearing of low-heeled shoes), and treatment may slow disease progression, thus preventing or delaying the onset of more serious symptoms and irreversible venous structural changes. In particular, compression treatment is the main conservative strategy of CVIs [7]. An alternative conservative approach consists in using vasoactive drugs (VADs) to manage symptoms and to decrease edema. Several studies highlight the beneficial effects of VADs for the management of symptoms of CVI patients [8]. Indeed, these drugs have shown a promising pharmacological efficacy profile by offering beneficial effects on CVI symptoms and quality of life due to their anti-inflammatory and anti-oxidant properties.

VADs are a heterogeneous group of drugs that includes synthetic and plant-derived substances [9], also used to improve compression therapy effects. Indeed, ESVS guidelines suggest a combination strategy with conservative therapies, in accordance with physicians and patient intention. Moreover, the ESVS guidelines reported a summary table with the effects of the main VADs on venous symptoms. 

Ruscus extracts [10], micronized purified flavonoid fraction (MPFF) [11], calcium dobesilate [12,13], horse chestnut extract [14], hydroxyethyl-rutosides [15], red vine leaf extract [16,17] and sulodexide [18] are the most widely used VADs in clinical practice.

In particular, MPFF (consisting of 90% diosmin and 10% concomitant active flavonoids, including hesperidin, linarin and isorhoifolin) is the most widely prescribed VAD in Europe [19] and is also available as an over-the-counter product in several countries. Other flavonoid-based drugs (Oxerutina (Venoruton^®^), ESSAVEN-Escin (Aventis^®^)) as well as dietary supplements, including Pycnogenol^®^, Antistax^®^ and Lenidase^®^, are used for CVI treatment.

**Figure 1:**
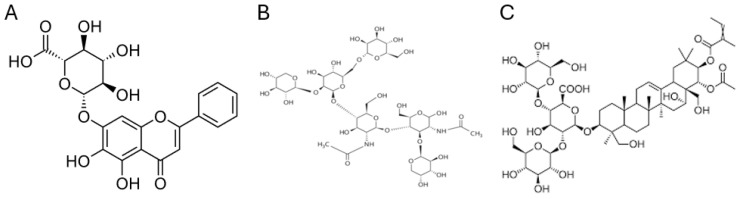
Lenidase^®^: Baicalin (**A**), Bromelain (**B**) and Escin (**C**) chemical structure.

Lenidase^®^ is a dietary supplement containing three flavonoids (*Scutellaria baicalensis* G. radix dry extract 200 mg, Baicalin 190 mg; Bromelain from *Ananas comosus* (L.) M. stipites dry extract 50 mg; *Aesculus hippocastanum* L. cortex 200 mg, Escin 60 mg) (Figure 1), which is able to improve microcirculation and drainage of body fluids.

In detail, baicalin is a natural flavonoid found in several plants, especially in the roots of *Scutellaria baicalensis*, a plant used in traditional Chinese medicine. Previous studies have already demonstrated baicalin’s anti-oxidant and anti-inflammatory effects, thanks to its ability in reducing pro-inflammatory cytokines and C-reactive protein (CRP) [20,21,22].

Bromelain is a proteolytic enzyme found in pineapple (*Ananas comosus*), mainly extracted from the stem and juice of the pineapple fruit. Bromelain plays anti-inflammatory, digestive and fibrinolytic effects, thus inhibiting inflammatory markers, such as Nuclear Factor kappa B (NF-κB), and reducing cyclooxygenase (COX)-2 and prostaglandin (PG) E-2 levels [23]. Moreover, some studies have shown its potential for the treatment of circulatory and venous diseases [24].

Escin is a natural chemical compound, found in the seeds and bark of the horse chestnut tree (*Aesculus hippocastanum*), used in traditional medicine for its anti-inflammatory, anti-oxidant and venotonic properties [20,21,25]. Escin acts on the microcirculation by reducing vascular permeability and increasing venous tone and venous return with consequent reduction of edema [26,27,28,29]. In fact, this natural product is used as dietary supplement to treat circulatory disorders, especially those related to CVI and capillary fragility, thus strengthening blood vessel walls and venous circulation, and reducing swelling, heaviness in the legs, and other symptoms associated with venous diseases.

Therefore, the aim of the present study was to evaluate the effects of a dietary supplement (Lenidase^®^) containing baicalin, escin and bromelin, in patients affected by CVI.

## 2. Results

Out of the total number of patients with CVI, 62 patients were selected: 30 (48.4%) and 32 (51.6%) were users and non-users, respectively.

Patients’ characteristics at baseline are reported in Table 1. No significant difference was observed for all considered variables between users and non-users at baseline (Table 1).

### 2.1. Efficacy of Dietary Supplement Treatment

#### 2.1.1. Primary Efficacy Outcome

The VCSS was evaluated as primary outcome at different timepoints. VCSS significantly decreased during the observation period in both groups (Table 2 and Table 3). However, the percentage reduction in VCSS was significantly higher in users than that observed in non-users at both T1 (median % change (Q1–Q3): −9.1 (−17.5/0.0) vs. −0.0 (0.0/0.0); *p* = 0.002) and T2 (median % change (Q1–Q3): −20.0 (−30.0/−9.1) vs. 0.0 (−15.3/−0.0); *p* = 0.019) (Table 4).

A significant improvement in SP and VI was observed in both groups, while Pain and INF improved in users, only (Table 2 and Table 3).

**Figure 2:**
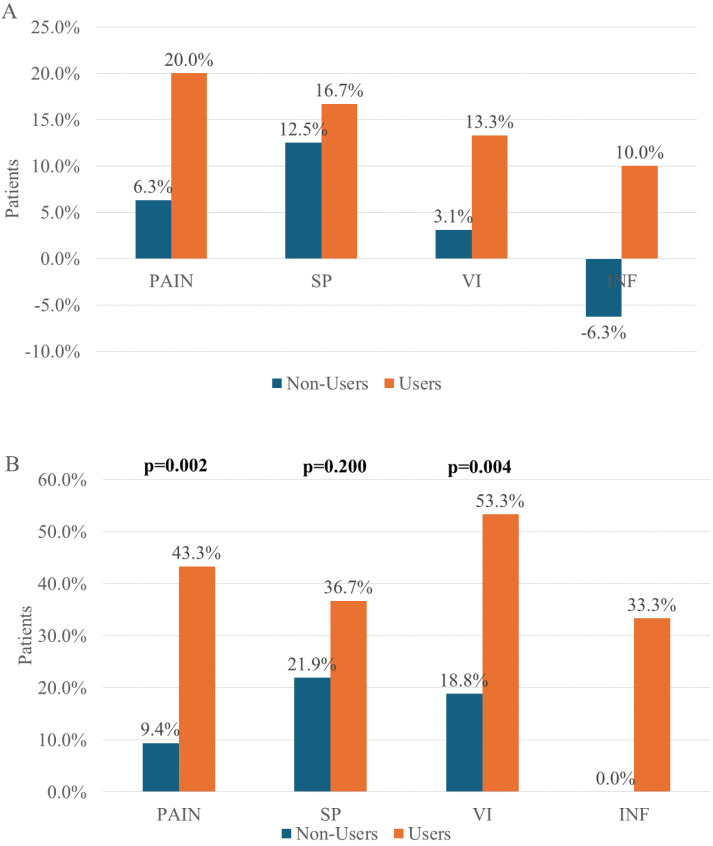
(%) Patients who had improved outcomes at T1 (**A**) and T2 (**B**). SP: skin pigmentation; VI: vessel induration; INF: inflammation.

In detail, among non-users, 2 (6.3%), patients experienced a worsening of clinical signs of inflammation at the 30-day follow up and no change was observed in the remaining patients, while inflammation reduced in 10% of users (Figure 2A). Moreover, the improvement of clinical signs of inflammation was observed only in the same two patients in the non-user group, after 90 days, while inflammation reduced in 33.3% of users (Figure 2B).

A reduction in pain was observed in 6.3% and 9.4% of non-users after 30 and 90 days of follow up, respectively. A significant decrease in pain was observed in users during the follow-up (20.0% and 43.3% of patients). Moreover, pain improvement was significantly higher in users than non-users in T2 (*p* = 0.002) (Figure 2B)

An improvement in SP stage was observed in 4 (12.5%) non-users and 5 (16.7%) users as well as in 7 (21.9%) non-users and 11 (36.7%) users after 30 days and 90 days, respectively. No difference was shown between groups at the end of the study also (*p* = 0.200) (Figure 2). Otherwise, users (*n* = 16; 53.3%) showed an improvement in VI stage compared to non-users (*n* = 6; 18.8%) (*p* = 0.004), as well as in the pain stage (users *n* = 13; 43.3% vs. non-users *n* = 3; 9.4%) (*p* = 0.002) (Figure 2).

Clinical evaluation of the criteria within the modified VCSS from baseline to T1 and T2 in users and non-users are described in Supplemental Figures S1–S4.

#### 2.1.2. Secondary Efficacy Outcomes

CRP levels (mg/dL) significantly decreased in both groups: from 2.8 (2.5–3.3) at baseline to 2.1 (2.0–2.9) at T1 and to 1.3 (1.0–1.5) at T2 (*p =*< 0.001) in non-users (Table 2); from 3.0 (2.5–3.5) at baseline to 2.0 (1.6–2.6) at T1 and to 1.0 (0.9–1.2) at T2 (*p* =< 0.001) in users (Table 3). However, a significant reduction was found in users compared to non-users at both T1 (median % change (Q1–Q3): −29.0 (−38.6/−22.0) vs. −15.9 (−22.9/−11.7); *p* < 0.001) and T2 (median % change (Q1–Q3): −64.5 (−73.6/−54.5) vs. 58.0 (−63.7/−54.5); *p* = 0.019) (Table 4).

No change in MC was observed in non-users (Table 2); conversely, MC value significantly decreased in users from 29.0 (26.5–30.0) at baseline to 28.5 (26.5–29.0) at T1 and 27.5 (26.0–28.5) at T2 (*p* < 0.001) (Table 3).

SBP values decreased in non-users from 127.5 (120.0–130.0) at T0 to 125.0 (120.0–130.0) at T2 (*p* = 0.032) and in users from 130.0 (120.0–135.0) at T0 to 120.0 (120.0–120.0) at T2 (*p* =< 0.001) (Table 2 and Table 3).

Furthermore, the SBP parameter significantly decreased from baseline to T2 in users compared to non-users (median % change (Q1–Q3): −7.7 (−11.1/0.0) vs. 0.0 (−7.1/0.0); *p* = 0.012) (Table 4).

On the contrary, no difference was observed in DBP between or within groups, at any time point. Similarly, no significant difference was detected in Cr values between users and non-users, at any time point in both groups (Table 2, Table 3 and Table 4).

## 3. Discussion

CVI has a significant socioeconomic impact, mainly due to the cost of the treatment of the sequelae caused and/or promoted by the same clinical condition. Both CVI and the related conditions may negatively modify the quality of life of the affected patients, especially in the advanced stages of the disease [30]. CVI development is related to age [31], sex, obesity, and family history [32,33]; in particular, clinical evidence has shown that the likelihood of developing CVI doubled in individuals who are overweight and in females, and increased up to 11 times in patients with strong familiarity [34]. Accordingly, in the present study patients affected by CVI were mainly elderly, female and overweight.

The approved standard of care is by using medical compression stockings which are easy to use, non-invasive and counteract the primary pathophysiological mechanism of the disease by mechanical venous compression and muscle function improvement. Compression therapy is effective for the treatment of leg ulcers [35,36] but may also be useful in CVI initial stages by relieving symptoms, such as edema and leg heaviness [37], although it is often not enough to relieve signs and symptoms related to inflammatory reaction.

Previous data demonstrated that dietary supplements could be useful in supporting CVI evolution and may reduce symptoms [38,39]. Moreover, the use of dietary supplements as well as natural origin products may improve the adherence to therapy, especially in patients commonly affected by comorbidities that need a polytherapy. Previous clinical data demonstrated the anti-inflammatory and anti-oxidant effects of dietary supplements, as well as their ability in reducing venous symptoms.

In this study the dietary supplement Lenidase^®^, consisting of escin, bromelain and baicalin, improved both VCSS and symptoms in “user” patients affected by CVI compared to “non-users” who underwent compression therapy only. Since the anti-inflammatory effects of baicalin were well-known and widely described, we hypothesized that VCSS improvement could be mainly ascribed to this natural compound. In fact, baicalin has been proven to decrease the serum levels of pro-inflammatory cytokines and CRP [40]. Accordingly, we found that CRP significantly decreased in “users” compared to “non-users”.

Furthermore, both baicalin and escin reduce PGE2 synthesis [20,21], as does bromelain, thus inducing significant anti-inflammatory effect [23]. The modulation of inflammatory mediators is closely related to pain [41], therefore the pain-relieving effect observed in patients treated with Lenidase^®^ could be attributed to all natural products. Patients who experienced an improvement in clinical signs of pain and inflammation were those who received the dietary supplement: a significant reduction was observed in “users” compared to “non-users” at T2.

Escin may act on the microcirculation by reducing vascular permeability and increasing venous tone and venous return with consequent edema reduction [29] and, in our study, an improvement in vessels elasticity was observed in patients receiving the dietary supplement, probably as a consequence of escin activity.

In addition to cell component involvement, molecular pathways, such as that of NF-κB which stimulates matrix metalloproteinase 9 (MMP-9), are also involved in fluid accumulation, including edema [42,43,44]. Both baicalin and bromelain may inhibit NF-κB; in fact, an anti-edema effect was observed in “user” patients and the combined anti-edema and anti-inflammatory effects of the natural products contained in Lenidase^®^ could explain the significant reduction in MC observed in patients treated with the dietary supplement compared to those receiving standard compression treatment alone.

Moreover, baicalin attenuates the vasoconstriction induced by Angiotensin II (Ang II) and attenuates Ang II-induced intracellular Ca^2+^ release [45]. Intracellular Ca^2+^ regulation in vascular smooth muscle as well as activation of KATP channels are implicated in the vasorelaxant effect of baicalin [46]. Therefore, the effect of baicalin could justify the improvement in SBP observed in the “users” group.

On the contrary, although an improvement in SP was observed in both groups, it was a not significant change, probably because of the great variability of SP degree found in CVI affected patients [47,48,49]. Although brown pigmentation of the skin has been reported to be due to the accumulation of hemosiderin or melanin, hemosiderin is believed to be the primary factor [47,48,49,50]. Tiwary et al. [50] observed pigmentation and eczema in 24% of cases and stated that the severity of the disease increased with the severity of the pigmentation. Moreover, according to a recent study, pigmentation (19.3%) was identified as one of the most common skin findings observed in patients with CVI, diabetes, hyperlipidemia, and thyroid disease [51]. In our study, 40.3% and 41.9% of patients had a severe and moderate SP score, respectively. An improvement in SP was observed following 90 days of treatment; however, the observation period could be too short to observe an additional improvement due to the dietary supplement use with respect to the standard treatment alone. No significant adverse events were recorded in both groups during the study period and none of the patients discontinued the treatment, thus suggesting the good safety profile of this dietary supplement.

## 4. Strengths and Weaknesses of the Study

Even if these findings are intriguing and suggest the efficacy of the dietary supplement as adjuvant therapy in CVI, several limits to our study should be considered.

The use of the modified VCSS, which considers only 4 of the 10 evaluation criteria, is a weakness of our study. However, each clinical criterion can be separately evaluated and the results confirm the effectiveness of the treatment on pain, inflammation and hardening of the vessels, although only few plasmatic inflammatory indicators (CRP) were recorded in the clinical records. Furthermore, we cannot attribute the observed effects to a single component or to a synergistic effect.

The sample size could also be considered a limit of this study, even if the analyses showed significant differences depending on the considered parameter. For sure, the results obtained in this preliminary study need to be further confirmed through the sample size.

## 5. Materials and Methods

### 5.1. Data Source

A retrospective cohort study was performed by using the computerized medical records of outpatient affected by CVI attending the Unit of Internal Medicine of the AOU “G. Martino” from 2019 to 2020. Following the approval by the local Ethical Committee of Messina University Hospital (Prot. 01-23 date 9 January 2023), the study has been carried out in accordance with the code of ethics of the World Medical Association (Declaration of Helsinki). The study participants were enrolled following informed consent collection. A patient encrypted code has been used to maintain anonymity.

The information relating to patients reported in computerized medical records included:

- Demographic and clinical characteristics, (age, sex, body mass index (BMI) malleolus circumference (MC)), data on diagnostic instrumental and laboratory tests (systolic (SBP) and diastolic (DBP) blood pressure, creatinine (Cr), CRP).

### 5.2. Study Population

Patients aged at least 18 years with a recorded diagnosis of CVI, with a clinical grade between C1 and C4 according to the CEAP classification, and on standard treatment with second class graduated compression stocking were selected.

Patients with arterial hypertension, ulcers, dermatitis, as well as patients who walk with the aid of a support and patients taking drugs within 15 days before starting treatment with the compression stock, were excluded.

The following clinical criteria were considered: pain, skin pigmentation (SP), inflammation (INF) and vessel induration (VI) within the VCSS calculated for each patient. The score assigned to each clinical criteria was related to the severity:

(0) Null
(1) Mild
(2) Moderate
(3) Severe

Patients using only compression stockings were defined as “non-users”, while patients treated with dietary supplements in combination with compression stockings were defined as “users”. All the variables considered were evaluated at baseline (T0), after 30 (T1) and 90 (T2) days. Dose of administration of the supplement was evaluated according to the severity of the symptoms as usually made in clinical practice, in particular, 1 capsule twice a day for 30 days and then 1 capsule per day up to 90 days in patients with mild-moderate symptoms; 2 capsules twice a day for 10 days followed by 1 capsule twice a day up to 90 days in patients with severe conditions.

### 5.3. Data Analysis

Demographic and clinical characteristics were evaluated by descriptive and comparative analyses between “non-user” and “user” groups and timepoints. In addition, the percentage variation of all outcomes from T0 to T1 and from T0 to T2 were analyzed. Categorial variables were calculated as absolute frequency and percentages, while continuous variables were calculated as medians and interquartile range (Q1–Q3). The Kolmogorov–Smirnov test was applied to evaluate the sample distribution; since some of the numeric variables were not normally distributed, a non-parametric approach was adopted. The Mann–Whitney U test for independent sample and two-tailed Pearson chi-squared test were carried out to compare continuous variables and categorical variables, respectively. Furthermore, the Kendall’s τ rank correlation test for ordinal variables and the Friedman test for continuous variables were used to compare paired data at different time points. A *p* value < 0.05 was considered as statistically significant. Statistical analysis was performed with SPSS version 23.0 (IBM Corp., SPSS Statistics; Chicago, IL, USA).

## 6. Conclusions

An adequate and preventive diagnosis of CVI, as well as the correct management of the disease, are needed.

Our study suggests that the use of the dietary supplements containing baicalin, bromelain and escin, in addition to the standard treatment, contributes to anti-inflammatory and anti-edema effects, thus enhancing therapeutic efficacy.

## Figures and Tables

**Table 1:** Demographic and clinical characteristics of CVI patients at baseline.

Variable: Median (IQR)	Non-Users *n* = 32	Users *n* = 30	*p*-Value
Age	73.5 (68.0–80.0)	71.0 (61.0–79.0)	0.296
Gender (F)	18 (56.3%)	19 (63.3%)	0.570
BMI (kg/m^2^)	27.0 (26.0–28.0)	26.2 (24.7–27.5)	0.072
SBP (mmHg)	127.5 (120.0–130.0)	130.0 (120.0–135.0)	0.624
DBP (mmHg)	70.0 (65.0–77.5)	70.0 (60.0–80.0)	0.715
Cr (mg/dL)	1.0 (0.9–1.2)	1.1 (0.8–1.2)	0.820
CRP (mg/dL)	2.8 (2.5–3.3)	3.0 (2.5–3.5)	0.454
MC (cm)	28.0 (27.5–28.5)	29.0 (26.5–30.0)	0.191
VCSS	9.0 (6.0–10.5)	9.0 (7.0–11.0)	0.864
SP Mild	6 (18.8)	5 (16.7)	0.896
SP Moderate	14 (43.8)	12 (40.0)	
SP Severe	12 (37.5)	13 (43.3)	
Pain Mild	6 (18.8)	6 (20.0)	0.991
Pain Moderate	14 (43.8)	13 (43.3)	
Pain Severe	12 (37.5)	11 (36.7)	
INF Mild	9 (28.1)	7 (23.3)	0.872
INF Moderate	13 (40.6)	12 (40.0)	
INF Severe	10 (31.3)	11 (36.7)	
VI Mild	5 (15.6)	6 (20.0)	0.725
VI Moderate	16 (50.0)	12 (40.0)	
VI Severe	11 (34.4)	12 (40.0)	

IQR: interquartile range; BMI: body mass index; SBP: systolic blood pressure; DBP: diastolic blood pressure; CRP: C-reactive Protein; MC: Malleolus Circumference; VCSS: Venous Clinical Severity Score; SP: skin pigmentation; INF: inflammation; VI: vessel induration.

**Table 2:** Clinical characteristics of non-users at T0, T1, T2.

Variables: Median (IQR)	Non-Users T0	Non-Users T1	Non-Users T2	*p*-Value
BMI (kg/m^2^)	27.0 (26.0–28.3)	26.9 (26.0–28.3)	26.9 (26.0–28.1)	0.002
SBP (mmHg)	127.5 (120.0–130.0)	125.0 (120.0–130.0)	125.0 (120.0–130.0)	0.032
DBP (mmHg)	70.0 (65.0–77.5)	70.0 (65.0–77.5)	70.0 (65.0–75.0)	0.988
Cr (mg/dL)	1.0 (0.9–1.2)	1.0 (0.8–1.2)	1.0 (0.8–1.2)	0.729
CRP (mg/dL)	2.8 (2.5–3.3)	2.1 (2.0–2.9)	1.3 (1.0–1.5)	<0.001
MC (cm)	28.0 (27.5–28.5)	28.0 (27.5–28.5)	28.0 (27.5–28.5)	0.050
VCSS	9.0 (6.0–10.5)	9.0 (6.0–10.5)	9.0 (5.0–10.0)	<0.001
SP Mild	6 (18.8)	8 (25.0)	10 (31.3)	0.005
SP Moderate	14 (43.8)	14 (43.8)	14 (43.8)	
SP Severe	12 (37.5)	10 (31.3)	8 (25.0)	
Pain Mild	6 (18.8)	7 (21.9)	7 (21.9)	0.097
Pain Moderate	14 (43.8)	14 (43.8)	15 (46.9)	
Pain Severe	12 (37.5)	11 (34.4)	10 (31.3)	
INF Mild	9 (28.1)	7 (21.9)	9 (28.1)	0.135
INF Moderate	13 (40.6)	15 (46.9)	13 (40.6)	
INF Severe	10 (31.3)	10 (31.3)	10 (31.3)	
VI Mild	5 (15.6)	6 (18.8)	10 (31.3)	0.006
VI Moderate	16 (50.0)	15 (46.9)	12 (37.5)	
VI Severe	11 (34.4)	11 (34.4)	10 (31.3)	

**Table 3:** Clinical characteristics of Users at T0, T1, T2.

Variables: Median (IQR)	Users T0	Users T1	Users T2	*p*-Value
BMI (kg/m^2^)	26.2 (24.7–27.5)	26.2 (24.7–27.5)	26.1 (24.7–27.3)	<0.001
SBP (mmHg)	130.0 (120.0–135.0)	125.0 (120.0–130.0)	120.0 (120.0–120.0)	<0.001
DBP (mmHg)	70.0 (60.0–80.0)	70.0 (60.0–80.0)	70.0 (60.0–80.0)	0.500
Cr (mg/dL)	1.1 (0.8–1.2)	1.1 (0.9–1.2)	1.1 (0.8–1.2)	0.132
CRP (mg/dL)	3.0 (2.5–3.5)	2.0 (1.6–2.6)	1.0 (0.9–1.2)	<0.001
MC (cm)	29.0 (26.5–30.0)	28.5 (26.5–29.0)	27.5 (26.0–28.5)	<0.001
VCSS	9.0 (7.0–11.0)	9.0 (5.0–10.0)	7.0 (4.0–9.0)	<0.001
SP Mild	5 (16.7)	9 (30.0)	13 (43.3)	<0.001
SP Moderate	12 (40.0)	12 (40.0)	10 (33.3)	
SP Severe	13 (43.3)	9 (30.0)	7 (23.3)	
Pain Mild	6 (20.0)	9 (30.0)	14 (46.7)	<0.001
Pain Moderate	13 (43.3)	13 (43.3)	10 (33.3)	
Pain Severe	11 (36.7)	8 (26.7)	6 (20.0)	
INF Mild	7 (23.3)	8 (26.7)	12 (40.0)	<0.001
INF Moderate	12 (40.0)	13 (43.3)	12 (40.0)	
INF Severe	11 (36.7)	9 (30.0)	6 (20.0)	
VI Mild	6 (20.0)	8 (26.7)	15 (50.0)	<0.001
VI Moderate	12 (40.0)	12 (40.0)	12 (40.0)	
VI Severe	12 (40.0)	10 (33.3)	3 (10.0)	

**Table 4:** Comparison of the percentage change between the 2 groups at T1 and T2.

Variable: Median (IQR)	Non-Users % Change T1	Users % Change T1	*p*-Value	Non-users % Change T2	Users % Change T2	*p*-Value
BMI	0.0 (0.0/0.0)	0.0 (0.0/0.0)	0.270	0.0 (0.0/0.0)	0.0 (−1.47/0.0)	0.076
SBD	0 (−7.1/0.0)	0 (−7.2/0.0)	0.390	0 (−7.1/0.0)	−7.7 (−11.1/0.0)	0.012
DBP	0.0 (−3.9/4.7)	0.0 (−1.4/12.9)	0.792	0.0 (−12.5/12.4)	0.0 (−7.2/9.5)	0.810
Cr	0.0 (−7.0/0.0)	0.0 (−8.3/0.0)	0.537	4.1 (−20.0/24.3)	0.0 (−14.3/0.0)	0.357
MC	0.0 (0.0/0.0)	−1.9 (−3.5/0.0)	<0.001	0.0 (0.0/0.0)	−5.0 (−7.2/−3.4)	<0.001
CRP	−15.9 (−22.9/−11.7)	−29.0 (−38.6/−22.0)	<0.001	−58.0 (−63.7/−54.5)	−64.5 (−73.6/−54.5)	0.019
VCSS	0.0 (0.0/0.0)	−9.1 (−17.5/0.0)	0.002	0.0 (−15.3/0.0)	−20.0 (−30.0/−9.1)	<0.001

## Data Availability

Data will be made available on request.

## Supplementary Materials

The following supporting information can be downloaded at: https://www.mdpi.com/article/10.3390/ph17060779/s1. Figure S1. Flowchart of the clinical evaluation change of pain stage in Users and Non—Users. Figure S2. Flowchart of the clinical evaluation change of vessels induration stage in Users and Non—Users. Figure S3. Flowchart of the clinical evaluation change of inflammation stage in Users and Non—Users. Figure S4. Flowchart of the clinical evaluation change of skin pigmentation stage in Users and Non—Users.
