# Supplementary material for: Effects of a Dietary Supplement Composed of Baicalin, Bromelain and Escin for Venous Chronic Insufficiency Treatment: Insights from a Retrospective Observational Study
Source: Pharmaceuticals (Basel). 2024 Jun 14;17(6):779. doi: 10.3390/ph17060779 (PMC11206508; doi:10.3390/ph17060779)

Figure S1. Flowchart of the clinical evaluation change of pain stage in Users and Non –Users.

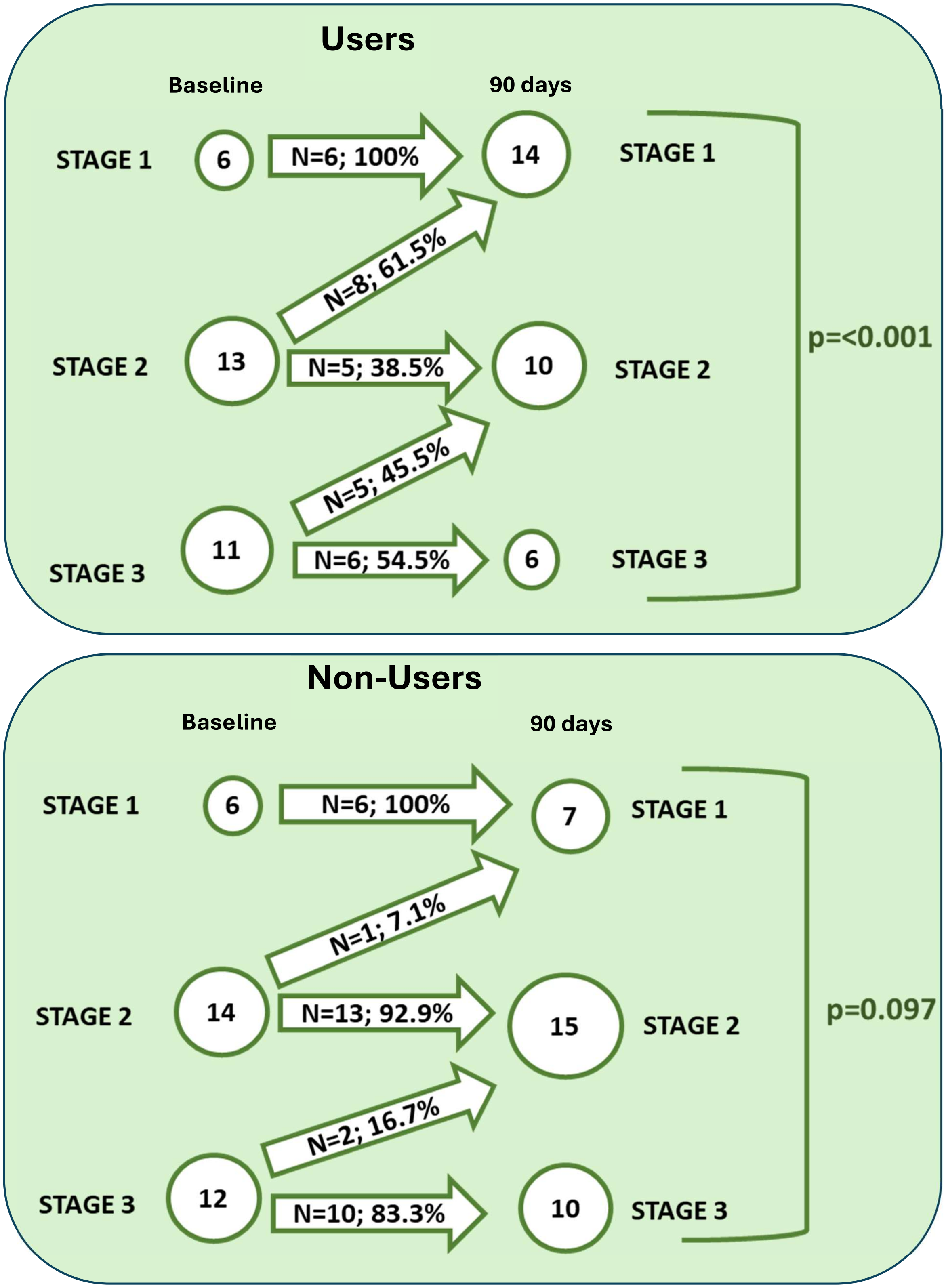

Figure S2. Flowchart of the clinical evaluation change of vessels induration stage in Users and Non –Users.

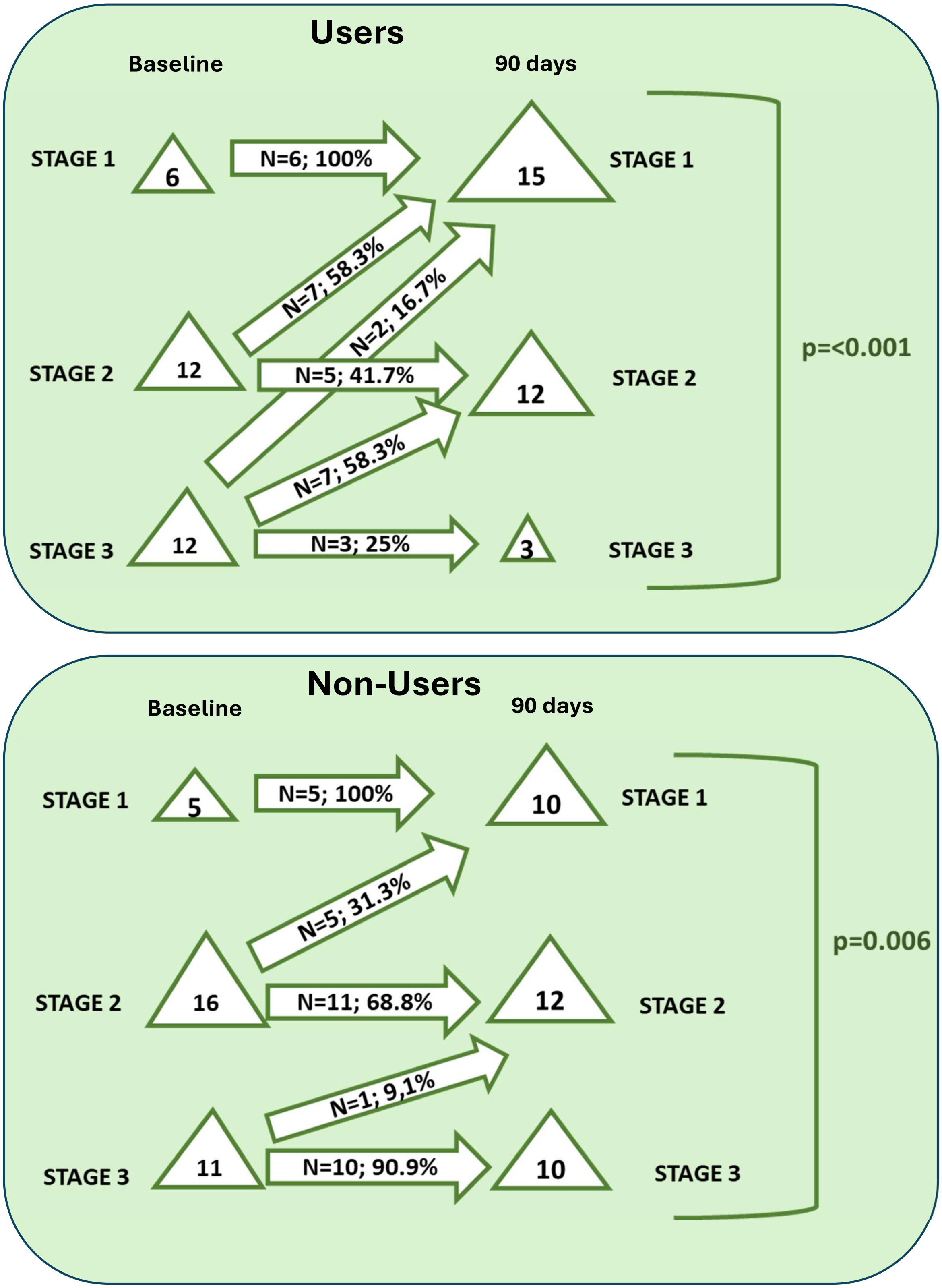

Figure S3. Flowchart of the clinical evaluation change of inflammation stage in Users and Non –Users.

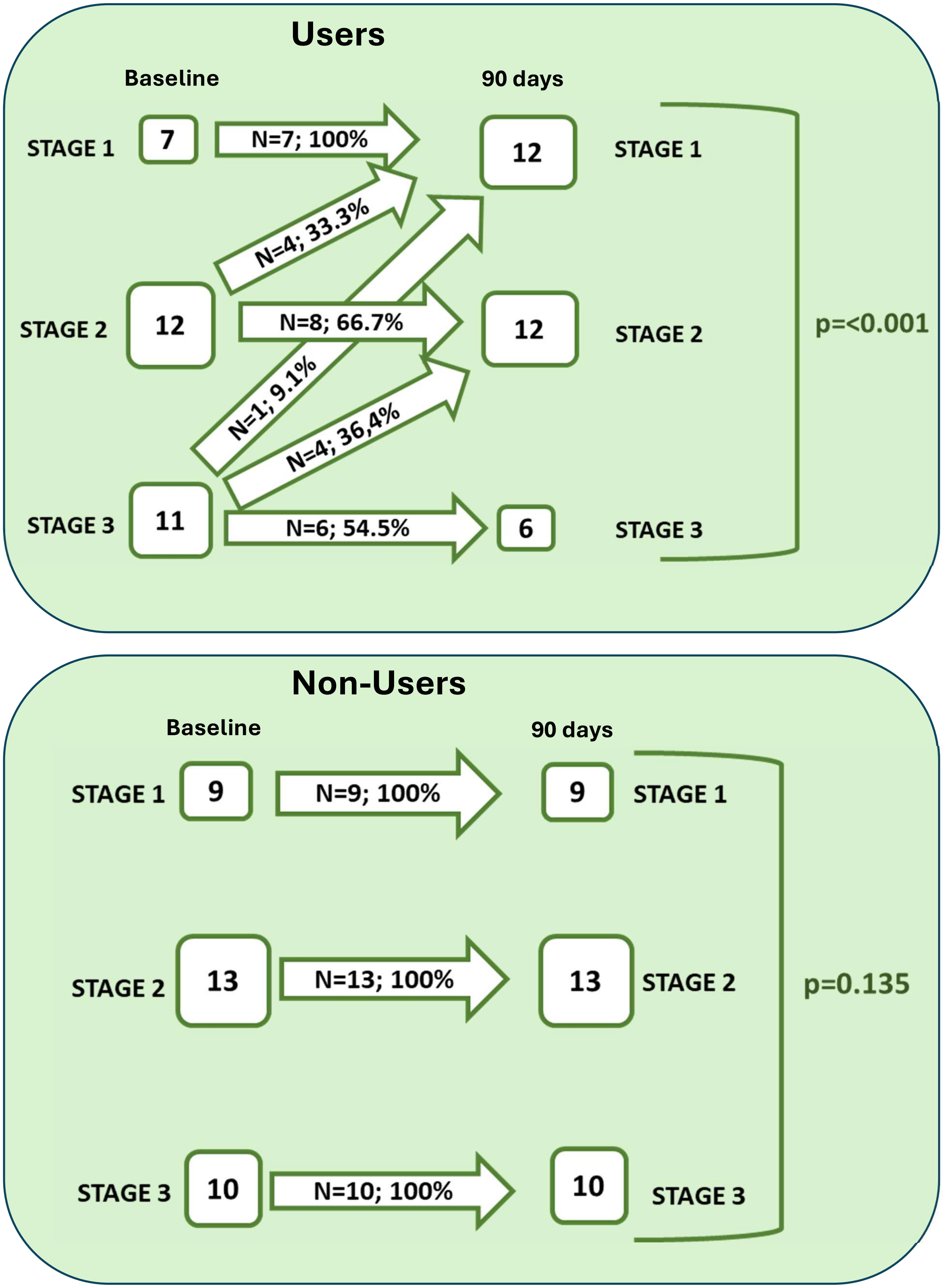

Figure S4. Flowchart of the clinical evaluation change of skin pigmentation stage in Users and Non –Users.

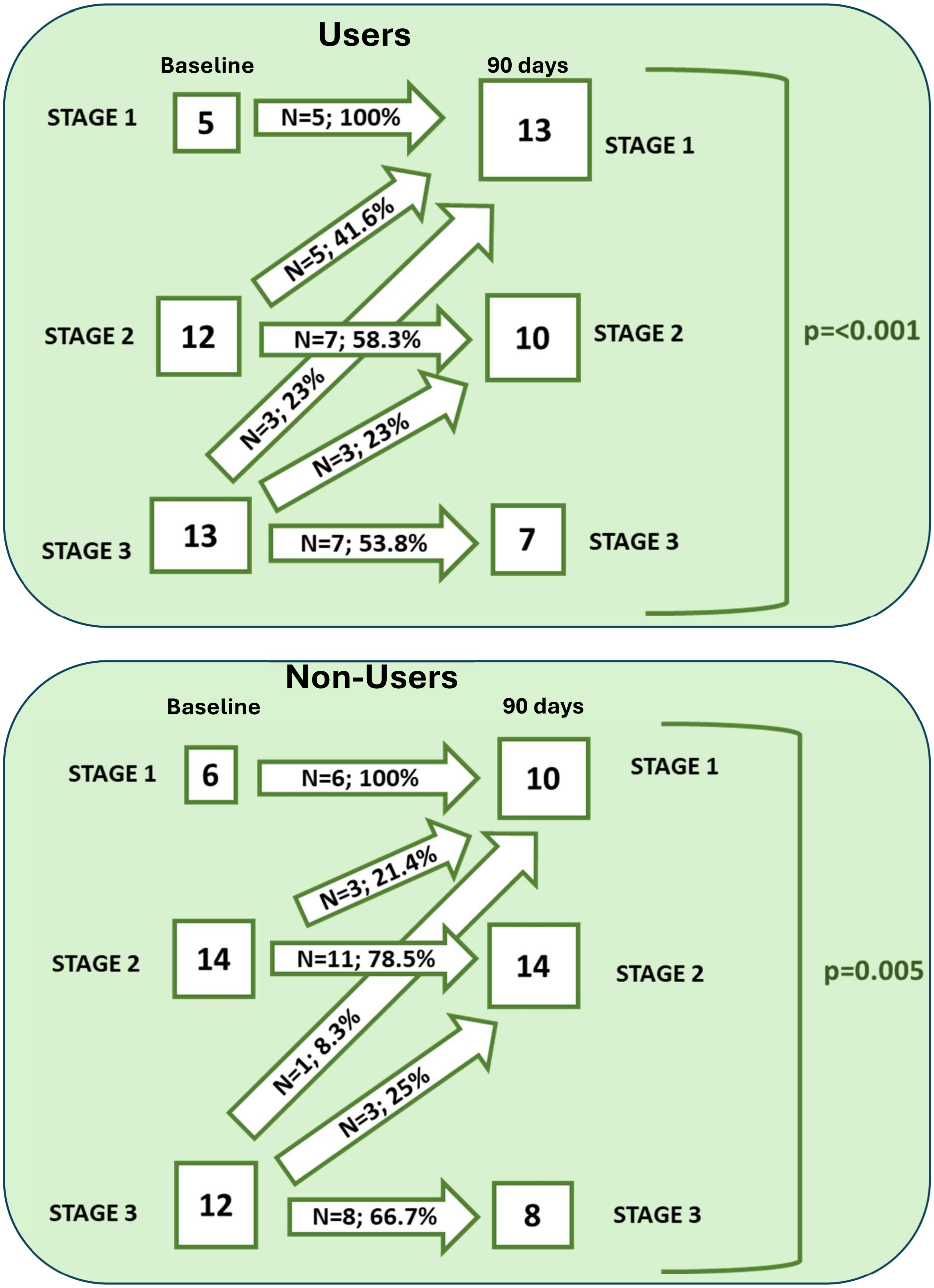

Supplement: Supplementary file 1 [file pharmaceuticals-17-00779-s001.zip › pharmaceuticals-2985861-supplementary.pdf]
